# Cognitive Performance Is Highly Stable over a 2-Year-Follow-Up in Chronic Kidney Disease Patients in a Dedicated Medical Environment

**DOI:** 10.1371/journal.pone.0166530

**Published:** 2016-11-11

**Authors:** Janine Gronewold, Olga Todica, Ulla K. Seidel, Michaela Volsek, Andreas Kribben, Heike Bruck, Dirk M. Hermann

**Affiliations:** 1 Department of Neurology, University Hospital Essen, University of Duisburg-Essen, Essen, Germany; 2 Department of Nephrology, University Hospital Essen, University of Duisburg-Essen, Essen, Germany; Kaohsiung Medical University Hospital, TAIWAN

## Abstract

**Background:**

As kidney and brain functions decline with aging, chronic kidney disease (CKD) and dementia are becoming increasing health burdens worldwide. Among the risk factors for cognitive impairment, CKD is increasingly recognized. The precise impact of CKD on the development of cognitive impairment is poorly understood.

**Methods:**

In the New Tools for the Prevention of Cardiovascular Disease in Chronic Kidney Disease (NT^CVD^) cohort, which was recruited in a dedicated nephrology department, we examined the 2-year course of cognitive performance in 120 patients (73 patients with CKD stages 3–5D, 47 control patients without CKD with similar vascular risk profile) using a comprehensive battery of 10 neuropsychological tests.

**Results:**

Kidney function, vascular risk factors and cognitive performance were highly stable both in CKD and control patients. The summary score of cognitive performance in CKD patients was very similar at baseline (z = -0.63±0.76) and follow-up (z = -0.54±0.79, p = 0.113), as was cognitive performance in control patients (z = -0.01±0.59 and 0.01±0.70, p = 0.862, at baseline and follow-up, respectively). Total serum cholesterol (199.6±36.0 and 186.0±32.9, p = 0.005 in controls; 194.4±46.1 and 181.2±41.2, p = 0.008 in CKD) and common carotid intima-media thickness (0.87±0.18 and 0.84±0.17, p = 0.351 in controls; 0.88±0.21 and 0.82±0.16, p = 0.002 in CKD) moderately but significantly decreased during the follow-up. In multivariable regression analyses, high age (β = -0.28, 95%CI = -0.48 to 0.08, p = 0.007) predicted decrease in cognitive performance.

**Conclusions:**

In this well-defined cohort receiving state-of-the-art therapy, cognitive performance did not decrease over 2 years. Our data emphasize the aspect of risk factor control, suggesting that dedicated medical care might prevent cognitive decline in CKD patients.

## Introduction

Chronic kidney disease (CKD) is a growing health problem worldwide with prevalence estimates ranging from 23.4–35.8% in persons aged 64 years or older [1]. Exhibiting a high load of vascular risk factors [2], CKD patients frequently reveal cognitive impairment in cross-sectional studies [3–6]. In an own cross-sectional analysis within the New Tools for the Prevention of Cardiovascular Disease in Chronic Kidney Disease (NT^CVD^) cohort that compared 119 CKD patients with 54 control patients without CKD but with similar vascular risk profile, deficits in memory, information processing, executive functions, language and visuoconstruction were noted in 19–39% of CKD patients [7]. High patient age, high HbA1c and high fibrinogen in blood predicted cognitive deficits, suggesting a role of disturbed glucose control and inflammation in the development of cognitive deficits [7].

Cross-sectional studies provide valuable insights into associations, but not into causal links between CKD and cognitive performance. Longitudinal studies, on the other hand, mostly used short screening tools like the Mini-Mental State Examination (MMSE) test or telephone interviews to assess cognitive performance [8–21]. These studies did not offer a consistent picture of the association between CKD and cognitive performance. So far, only two studies used a comprehensive neuropsychological test battery to measure cognitive impairment and cognitive decline: In the residential care facility-based prospective Rush Memory and Aging Project cohort that included 886 participants, Buchman et al. (2009) revealed that lower baseline kidney function indicated by lower estimated glomerular filtration rate (eGFR) was associated with annual decline in a summary score of global cognition within 3.4±1.4 years follow-up [22]. Conversely, in the community-based Maine-Syracuse longitudinal study that included 590 participants, Davey et al. (2013) did not identify an association between baseline eGFR and change in cognitive function but only between change in eGFR and change in global cognitive function within 5 years of follow-up [23].

All above-mentioned studies examined cohorts of elderly subjects, which were recruited outside hospitals and which only partly exhibited CKD. These studies were not explicitly designed to analyze links between cognition and CKD. So far, only one study systematically compared 62 elderly CKD patients aged 78.1±3.6 years with 63 subjects without CKD with similar age and vascular risk profile that were recruited via a residential care facility, showing similar progression of cognitive deficits evaluated by the MMSE test and the clinical dementia rating (CDR) scale within 3 years follow-up in subjects with and without CKD [24]. In CKD patients, the MMSE score decreased by 4.7 points within the observation period [24]. Again, this study lacked a more thorough neuropsychological examination. Following our previous cross-sectional analyses in CKD patients, in which we evaluated links between vascular risk factors, cognition, emotion and quality of life [7, 25], we in the meantime completed a two-year follow-up examination in the NT^CVD^ cohort. We now used this two-year follow-up to evaluate links between CKD and the longitudinal course of cognitive performance. Since the NT^CVD^ cohort was recruited in a dedicated university department of nephrology, this cohort represents a sample of well-controlled CKD patients as well as control patients without CKD but with similar vascular risk profile receiving optimized treatment for CKD and for associated vascular risk factors and diseases.

## Materials and Methods

### Study cohort

**Fig 1:**
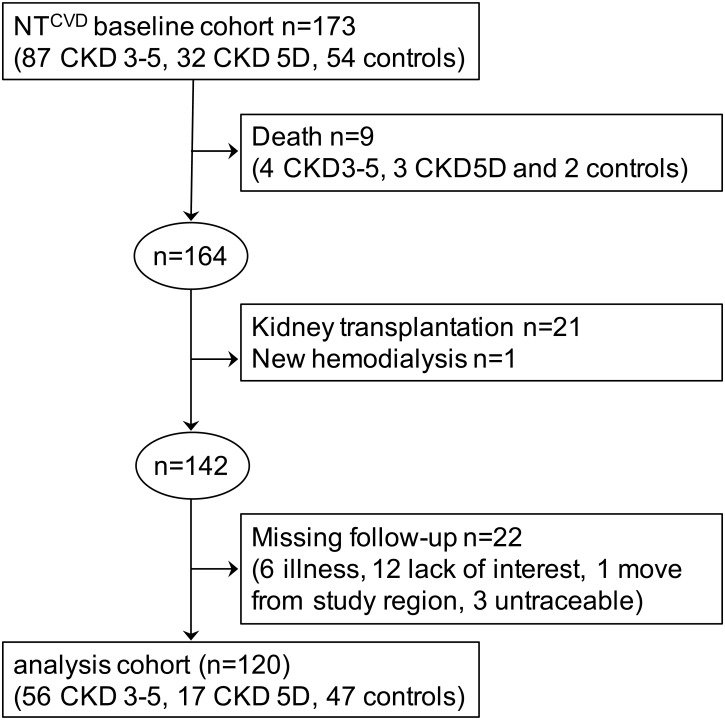
Flow chart of the NT^CVD^ 2-year follow-up study. Numbers of patients included and excluded in the follow-up analysis are shown. CKD, chronic kidney disease. NT^CVD^, New Tools for the Prevention of Cardiovascular Disease in Chronic Kidney Disease.

The NT^CVD^ study prospectively analyzed the effects of risk factors and markers of cardiovascular disease in patients with CKD [26]. In October 2008, 87 patients with CKD3-5 without hemodialysis, 32 CKD patients on hemodialysis (CKD5D) and 54 control patients with vascular risk factors but without CKD according to the Kidney Disease Outcomes Quality Initiative classification [27] were recruited at the Department of Nephrology of the University Hospital Essen with the help of local collaborating physicians and received detailed neuropsychological examinations [7, 25]. The study was approved by the ethical committee of the University Duisburg-Essen and all subjects gave written informed consent. Of the 173 patients enrolled at baseline, 9 patients (4 CKD3-5, 3 CKD5D and 2 controls) died between baseline and the 2-year follow-up, on which this longitudinal analysis is based. Of the remaining 164 patients, 30 patients underwent kidney transplantation and one patient newly required hemodialysis. These patients were excluded from the present analysis. Of the remaining 133 patients, 22 patients (16.5%) could not be examined at follow-up for the following reasons: 6 too ill, 12 no interest, 1 moved away from study region and 3 untraceable. Consequently, 120 patients (83.5% of 133 patients still available) were included into the present analysis (56 CKD3-5, 17 CKD5D and 47 control patients). A flow chart of the patient inclusion and exclusion is presented in Fig 1.

### Medical history and patient examination

Patient examination has been described in detail before [7, 25, 28]. At the baseline examination and the 2-year follow-up, all patients underwent a standardized interview to collect socio-demographic and medical history data, followed by a physical and neuropsychological examination as well as laboratory tests. Medical history included information about the onset, etiology and treatment of CKD, known vascular risk factors, namely arterial hypertension, dyslipidemia, diabetes, and current smoking (defined as smoking during the last 6 months), and vascular diseases (coronary heart disease (CHD), stroke/transient ischemic attack (TIA), peripheral artery disease (PAD)), and current medications. Level of education was assessed categorically as none, secondary school, baccalaureate or university degree as well as continuously as years of school education.

Blood and urine samples were collected to evaluate CKD stage and vascular risk profile in addition to routine blood parameters. Total cholesterol, low-density lipoprotein (LDL) cholesterol, high-density lipoprotein (HDL) cholesterol, triglycerides, glycated hemoglobin (HbA1c), glucose, creatinine, hemoglobin, potassium and fibrinogen were measured in peripheral blood using standard laboratory techniques, and urine creatinine, urea and proteins were evaluated by nephelometry at the central laboratory of the University Hospital Essen. eGFR was calculated using the modification-of-diet-in-renal-disease (MDRD) formula. Standardized hip and waist circumference, height and weight were measured. Standardized height and weight measurements were used for calculating the body-mass index (BMI).

Noninvasive tests included the measurement of systolic and diastolic blood pressure. Arterial hypertension was defined as systolic blood pressure ≥140 mmHg, diastolic blood pressure ≥90 mmHg, or prescription of antihypertensive drugs. Diabetes was coded in cases of physician–diagnosed diabetes, blood glucose ≥200 mg/dl or fasting glucose of ≥126 mg/dl, or prescription of antidiabetic medication. Dyslipidemia was defined as total cholesterol ≥240 mg/dl or LDL cholesterol ≥160 mg/dl, or prescription of lipid-lowering medication. Intima-media thickness (IMT) of both common carotid arteries and ankle-brachial index (ABI) were determined, as described before [7].

### Neuropsychological examination

The neuropsychological test battery comprised 10 standardized tests that included (a) the revised Wechsler memory scale (subtests digit and block span, forward and backward; assessment of verbal and spatial short-term and working memory) [29], (b) the trail making test part A (assessment of information processing speed) [30], (c) the trail making test part B (assessment of cognitive flexibility) [30], (d) the Stroop test (assessment of cognitive interference) [31], (e) the Regensburg word fluency test (subtests ‘animals’ and ‘s-words’; assessment of semantical and lexical word fluency) [32] and (f) the Rey–Osterrieth complex figure test (copy task, assessment of visuoconstructive function) [33]. To control for depression and anxiety level, the Hospital Anxiety and Depression Scale was applied [34].

In order to obtain a score for global cognition, z-scores were calculated for all 10 neuropsychological tests on the basis of norm values generated in the control group [7].

Thus, the mean value of control patients at the baseline examination was subtracted from each individual CKD patient score and divided by the standard deviation (SD) of control patients at baseline. A summary score of global cognitive performance was formed by computing mean values of these z-scores. For the TMT part A/B and Stroop tests, scores were reversed because lower scores indicate better performance.

Besides the summary score, cognitive subdomains were computed, namely memory (digit and block span, forward and backward), information processing speed (TMT part A), executive function (TMT part B, Stroop), language (word fluency ‘animals’ and ‘s-words’), and visuoconstructive function (Rey–Osterrieth complex figure copy task), by computing mean values of z-scores of the respective tests. Change in cognition was evaluated by subtracting the baseline from the follow-up performance, thus positive values indicate improvement in performance while negative values indicate cognitive decline.

### Statistical analysis

Continuous data are presented as mean±SD values for normally distributed data and as median (Q1;Q3) for non-normally distributed data, categorical data as counts (%). To evaluate the effect of CKD and time on continuous medical and neuropsychological data, a mixed-design ANOVA with the between-subjects factor group (CKD3-5D vs controls) and the within-subjects factor time (baseline vs 2-year follow-up) with post-hoc unpaired or paired t-tests, respectively, for normally distributed data or Mann-Whitney tests and Wilcoxon signed-rank tests, respectively, for non-normally distributed data was performed. Categorical data were analyzed with Chi-square or Fisher’s exact tests. To evaluate predictors of change in cognition, univariate and multivariable linear regressions (forced entry method) were calculated. The factors age, sex, years of school education, eGFR, hemoglobin, potassium, urine urea, urine total protein, urine albumin, systolic blood pressure, total cholesterol, HDL cholesterol, LDL cholesterol, HbA1c, smoking, BMI, fibrinogen, IMT, ABI, cardiovascular diseases (CHD, stroke/TIA, PAD), depression, anxiety, antidiabetics, antihypertensives, lipid-lowering drugs and baseline cognitive performance were first inserted unadjusted into these regressions. For urine urea, urine albumin and urine total protein, the natural logarithm was used to normalize these right-skewed variables. In case of eGFR, the value 7.5 ml/min/1.73 m^2^ was used for hemodialysis patients which represents half the cut-off for CKD5 patients, because eGFR is not valid in hemodialysis patients. In a next step we analyzed the influence on cognitive performance of (a) age, sex, education and eGFR (model 1), (b) age, sex, education, eGFR and baseline cognition (model 2), (c) age, sex, education, eGFR, vascular risk factors (systolic blood pressure, HbA1c, total cholesterol), fibrinogen and baseline cognition (model 3), (d) age, sex, education, eGFR, the subclinical atherosclerosis marker IMT and baseline cognition (model 4) and (e) age, sex, education, eGFR, vascular diseases (CHD, stroke/TIA, PAD) and baseline cognition (model 5). The remaining variables were not included into multivariable analyses, since they did not reveal associations with change in cognitive performance in univariate analyses (not shown). In order to analyze the influence of change of predictors from baseline to follow-up on change of cognition, we repeated the above-mentioned regressions using change values instead of baseline values. Cases with missing values were excluded listwise from the analysis. P-values <0.05 indicate statistical significance. All statistics were performed using SPSS19 for Windows (SPSS, Chicago, IL, U.S.A.).

## Results

### Study cohort

The characteristics of the total study cohort including 120 patients with complete neuropsychological data at baseline and follow-up (73 CKD and 47 control patients) are summarized in Table 1. The 53 patients (46 CKD and 7 control patients) who had only baseline and no follow-up investigation due to reasons shown in Fig 1 and were thus excluded from the present analysis, were significantly younger (57.7±16.8 vs 63.6±12.3 years, p = 0.020), had lower BMI (26.5±5.1 vs 28.2±5.0 kg/m^2^, p = 0.038), lower eGFR (14(7.5;43.3) vs 53.5(28.3;65.0) ml/min/1.73m^2^, p<0.001), higher creatinine (3.5(1.6;5.5) vs 1.3(1.1;2.3) mg/dl, p<0.001), lower hemoglobin (12.0±1.8 vs 13.5±1.5 g/dl, p<0.001), higher potassium (4.7±0.7 vs 4.6±0.6 mmol/l, p = 0.044), higher fibrinogen (435.5±120.1 vs 374.1±101.5 mg/dl, p = 0.002), higher urine albumin (784.1(114.1;1749.0) vs 35.2(17.9;128.9) mg/g creatinine, p<0.001) and higher urine protein (1307.7(611.0;3369.8) vs 187.3(124.5;389.8) mg/g creatinine, p<0.001) compared with the 120 patients with complete data at baseline and follow-up included in the present analysis.

**Table 1:** Charateristics of Control Patients and CKD Patients at Baseline and Follow-up.

	Controls (n = 47)			CKD stages 3-5D (n = 73)				
	Baseline	Follow-up	p-values compared with baseline	Baseline	Follow-up	p-value compared with baseline	p-value controls vs CKD patients baseline	p-value controls vs CKD patients follow-up
Age (years)	62.6±10.2	64.7±10.2		64.3±13.6	66.4±13.6		0.424	
Gender (male)	35(74.5)	35(74.5)		42(57.5)	42(57.5)		0.079	
Highest academic degree								
No degree	1(2.1)	1(2.1)		5(6.8)	5(6.8)		0.114	
Secondary School	34(72.3)	34(72.3)		59(80.8)	59(80.8)			
Baccalaureate	6(12.8)	6(12.8)		7(9.6)	7(9.6)			
University degree	6(12.8)	6(12.8)		2(2.7)	2(2.7)			
Arterial hypertension	41(89.1)	42(89.4)	0.999	68(94.4)	69(94.5)	0.999	0.309	0.311
Dyslipidemia	36(78.3)	40(85.1)	0.289	60(83.3)	61(83.6)	0.999	0.629	0.999
Diabetes	14(35.9)	16(34.8)	0.999	30(44.8)	35(50.0)	0.625	0.418	0.128
Smoking	7(15.2)	7(14.9)	0.999	7(9.7)	5(6.8)	0.500	0.394	0.231
BMI (kg/m^2^)	28.3±5.1	27.8±4.8	0.053	28.1±4.9	28.0±5.0	0.789	0.849	0.838
Waist circumference (cm)	100.7±13.6	101.9±12.3	0.896	104.3±14.4	102.3±14.4	0.399	0.231	0.895
Coronary heart disease	18(39.1)	21(45.7)	0.250	25(35.2)	26(36.6)	0.999	0.698	0.342
Myocardial infarction	8(17.4)	9(19.6)	0.999	7(10.0)	7(10.0)	0.999	0.269	0.173
Peripheral artery disease	2(4.7)	3(7.0)	0.999	7(10.3)	8(11.8)	0.999	0.478	0.525
Stroke/transient ischemic attack	0	0	0.999	4(5.7)	4(5.7)	0.999	0.216	0.216
Systolic blood pressure (mmHg)	142.4±22.3	137.4±17.5	0.112	141.8±26.4	141.3±25.5	0.969	0.896	0.343
Diastolic blood pressure (mmHg)	76.4±11.4	77.0±9.9	0.327	71.0±11.6	72.1±11.1	0.240	0.014	0.021
Total cholesterol (mg/dl)	199.6±36.0	186.0±32.9	**0.005**	194.4±46.1	181.2±41.2	**0.008**	0.512	0.508
LDL cholesterol (mg/dl)	118.5±27.1	113.2±26.1	0.145	106.4±35.6	100.1±32.5	0.138	**0.049**	**0.018**
HDL cholesterol (mg/dl)	52.0±14.8	51.0±15.2	0.315	53.6±19.7	51.4±18.9	**0.024**	0.640	0.914
Triglycerides (mg/dl)	131.0 (88.0;184.0)	124.0(81.0;163.0)	0.106	139.5(83.3;203.0)	124.5(90.3;203.8)	0.809	0.958	0.427
HbA1c (%)	6.14±1.02	6.16±1.09	0.624	6.18±1.00	6.30±1.05	**0.047**	0.815	0.481
Glucose (mg/dl)	113.6±30.1	109.4±28.8	0.062	119.4±43.0	114.2±45.5	0.103	0.386	0.479
eGFR (ml/min/1.73m^2^)	68.0(64.0;74.0)	71.0(61.0;78.0)	0.611	35.0(14.0;50.5)	33.0(14.0;50.5)	0.127	**<0.001**	**<0.001**
Creatinine (mg/dl)	1.1(1.0;1.2)	1.1(0.9;1.2)	0.753	1.9(1.3;3.6)	2.0(1.3;3.7)	0.159	**<0.001**	**<0.001**
Hemoglobin (g/dl)	14.2±1.2	14.1±1.4	0.665	13.0±1.5	12.8±1.6	0.297	**<0.001**	**<0.001**
Potassium (mmol/l)	4.2±0.3	4.2±0.3	0.935	4.6±0.7	4.7±0.7	0.186	**<0.001**	**<0.001**
Fibrinogen (mg/dl)	332.9±66.4	328.9±73.4	0.826	401.3±111.6	391.3±108.3	0.223	**<0.001**	**<0.001**
IMT (mm)	0.87±0.18	0.84±0.17	0.351	0.88±0.21	0.82±0.16	0.002	0.735	0.418
ABI	1.09±0.17	1.10±0.15	0.602	1.07±0.25	1.08±0.24	0.704	0.683	0.694
Urine albumin (mg/g creatinine)	25.5(15.3;41.5)	18.4(9.5;40.3)	**0.015**	49.1(22.7;444.1)	42.5(21.8;654.2)	0.775	**<0.001**	**<0.001**
Urine protein (mg/g creatinine)	136.9(104.1;222.7)	150.1(100.7,183.9)	0.666	244.1(140.5;1106.8)	240.5(152.8;1216.0)	0.368	**<0.001**	**<0.001**
Antidiabetics	11(23.4)	14(29.8)	0.250	25(34.2)	27(37.0)	0.625	0.206	0.438
Antihypertensives	41(87.2)	43(91.5)	0.625	67(91.8)	65(89.0)	0.625	0.536	0.763
Lipid-lowering medication	23(48.9)	27(57.4)	0.125	36(49.3)	38(52.1)	0.687	0.968	0.579
Antiplatelets	23(48.9)	25(53.2)	0.687	41(56.2)	45(61.6)	0.503	0.459	0.448

Data are means±SD for normally distributed continuous data or median(Q1;Q3) for non-normally distributed continuous data, categorical data are presented as n(%). Statistical comparisons were done as appropriate with tests described in the materials and methods section. P-values <0.05 are shown in bold. ABI, ankle-brachial index; BMI, body-mass index; CKD, chronic kidney disease; eGFR, estimated glomerular filtration rate; HbA1c, glycated hemoglobin; HDL, high-density lipoprotein; IMT, intima-media thickness of common carotid artery; LDL, low-density lipoprotein.

The mean age of those CKD patients enrolled was 64.3±13.6 years (57.5% males). Age and sex distribution did not significantly differ from control patients. As expected, CKD patients had lower eGFR, lower hemoglobin, a higher blood creatinine, potassium and fibrinogen and higher urine albumin and urine protein than control patients. LDL cholesterol and diastolic blood pressure were also lower in CKD patients than in control patients. All other variables did not differ between the CKD and the control group. During the 2-year follow-up, eGFR remained very stable in CKD patients and controls. A significant decline in eGFR of >3 mL/min/1.73 m²/year was noted in 16.4% of CKD and 25.5% of control patients. In line with the tight control of CKD and comorbid risk factors, blood total cholesterol moderately decreased in CKD and in control patients, as did HDL cholesterol and intima-media thickness of the common carotid artery (IMT) in CKD patients. Interestingly, HbA1c slightly increased in CKD patients (both in CKD stages 3–5 and stage 5D), while blood glucose remained unchanged. There was no significant interaction between time and group for none of the variables of interest. As a consequence of the intensified patient treatment, a large percentage of subjects in both groups received antidiabetic, antihypertensive, lipid-lowering and antiplatelet drugs.

### Cognitive performance

Results of the ten neuropsychological tests we used are presented in Table 2. Results for the summary score of global cognition, which comprised all ten tests, and cognitive domains are shown in Table 3. The participants who had only baseline and no follow-up investigation and were thus excluded from the present analysis did not significantly differ from the participants with complete data at baseline and follow-up included in the present analysis (not shown). As previously reported for the cross-sectional analysis [7], those CKD patients enrolled into the present analysis revealed significantly worse performance than controls in all tests except for the forward condition of the block span. In the longitudinal analysis however, CKD patients showed a similar course of cognitive performance compared to control patients. Notably in individual tests assessing working memory (block span backward), interference (Stroop test) and word fluency (‘animals’ and ‘s-words’), CKD patients even improved during the follow-up, which may reflect a consequence of test sophistication and practice effects. The summary score of global cognition and cognitive domains did not reveal any changes during the follow-up, except for language, which improved in CKD patients.

**Table 2:** Raw Values of Neuropsychological Tests of Control Patients and CKD patients at Baseline and Follow-up.

	Controls (n = 47)			CKD stages 3-5D (n = 73)				
	Baseline	Follow-up	p-value compared with baseline	Baseline	Follow-up	p-value compared with baseline	p-value controls vs CKD baseline	p-value controls vs CKD follow-up
Digit span, forward (correct responses)	7.9±1.8	7.6±1.7	0.261	6.7±1.6	6.6±1.8	0.716	**<0.001**	**0.002**
Digit span, backward (correct responses)	6.3±1.7	6.5±1.4	0.390	5.5±1.7	5.5±1.7	0.634	**0.017**	**0.001**
Block span, forward (correct responses)	7.3±1.6	7.5±1.6	0.479	7.7±1.5	7.5±1.6	0.268	0.120	0.934
Block span, backward (correct responses)	7.0±1.5	7.0±1.8	0.999	6.0±1.7	6.7±1.7	**0.001**	**0.001**	0.335
TMT part A (seconds)	38.8±17.0	39.8±14.1	0.861	49.3±23.3	50.4±27.1	0.723	**0.010**	**0.006**
TMT part B (seconds)	92.2±35.8	94.6±47.8	0.364	129.0±58.0	137.1±63.7	0.073	**<0.001**	**<0.001**
Stroop test, interference (seconds)	89.7±17.4	85.8±11.8	0.109	104.0±31.4	98.6±25.2	**0.028**	**0.002**	**0.001**
Word fluency, 'animals' (correct responses)	31.1±8.5	33.0±9.0	0.088	27.6±8.0	29.2±8.9	**0.028**	**0.027**	**0.028**
Word fluency, 's-words' (correct responses)	20.7±6.5	20.4±6.3	0.580	15.5±6.5	16.9±6.8	**0.044**	**<0.001**	**0.005**
Rey-Osterrieth Complex Figure Test (raw value)	32.9±2.2	32.3±3.7	0.610	30.5±4.9	31.1±3.5	0.648	**<0.001**	0.069
HADS, depression scale (score)	5.0(2.0;6.5)	3.0(2.0;6.0)	0.094	4.0(2.0;7.5)	4.0(1.0;8.0)	0.158	0.961	0.769
HADS, anxiety scale (score)	5.0(3.0;7.5)	4.0(2.75;6.0)	**0.011**	5.0(2.0;7.5)	4.0(2.0;8.0)	**0.044**	0.749	0.879

Data are means ± SD for normally distributed continuous data or median (Q1;Q3) for non-normally distributed continuous data. Statistical comparisons were done as appropriate with tests described in the materials and methods section. P-values <0.05 are shown in bold. CKD, chronic kidney disease; HADS, hospital anxiety and depression scale; TMT, trail-making test.

**Table 3:** z-values of Global Cognition and Cognitive Domains in Controls and CKD patients at Baseline and Follow-up.

	Controls (n = 47)			CKD stages 3-5D (n = 73)				
	Baseline	Follow-up	p-value compared with baseline	Baseline	Follow-up	p-value compared with baseline	p-value controls vs CKD baseline	p-value controls vs CKD follow-up
Global cognitive performance	0.00±0.59	0.00±0.70	0.862	-0.63±0.76	-0.54±0.79	0.113	**<0.001**	**<0.001**
Memory	0.00±0.64	0.03±0.73	0.742	-0.38±0.68	-0.32±0.75	0.445	**0.003**	**0.013**
Information processing speed	0.00±0.99	-0.06±0.83	0.861	-0.61±1.36	-0.68±1.59	0.723	**0.010**	**0.006**
Executive function	-0.03±0.93	0.01±1.21	0.693	-0.92±1.38	-0.96±1.37	0.827	**<0.001**	**<0.001**
Language	0.00±0.87	0.07±0.89	0.459	-0.61±0.85	-0.41±0.94	**0.008**	**<0.001**	**0.006**
Visuo-construction	0.00±1.00	-0.26±1.72	0.610	-1.12±2.28	-0.83±1.62	0.648	**<0.001**	0.069

Data are mean z-values ± SD. Statistical comparisons were done as appropriate with tests described in the materials and methods section. P-values <0.05 are shown in bold. Global cognitive performance: all neuropsychological tests; memory: digit and block span (forwards and backwards); information processing speed: trail-making test, part A; executive function: Stroop (interference) and trail-making test, part B; language: Regensburger Wortflüssigkeitstest ('animals' and 's-words'); visuo-construction: Rey-Osterrieth Complex Figure (copy task). CKD, chronic kidney disease.

In view of the absence of cognitive decline, we evaluated the statistical power of this observation in comparison to data presented in the literature. The only study hitherto reporting quantitative changes of cognitive performance in a longitudinal study using more detailed neuropsychological testing was that of Davey et al. [23], who described a decline of global cognitive performance of 0.075 z-values over 5 years, equivalent to a decrease of 0.03 z-values over 2 years. Based on the data presented in Table 3 we observed a change of global cognitive performance of 0.06 ± 0.45 z-values over 2 years in the total cohort. According to statistical power calculations, our study was suitable to detect the absence of cognitive deterioration with a statistical power (1-β) of 70.7% with an error probability (α-value) of 5% in a 120 patient cohort.

### Predictors for change in cognition

To evaluate predictors of cognitive changes within the 2-year follow-up, univariate and multivariable linear regressions were computed using the difference value of the summary score of global cognitive performance at follow-up and baseline (follow-up minus baseline, Tables 4 and 5). In univariate regressions, high IMT (β = -0.18, 95% CI = -0.36 to -0.01, p = 0.049) and high baseline cognitive performance (β = -0.21, 95% CI = -0.39 to -0.03, p = 0.023) predicted decrease in global cognitive performance, while all other variables did not have a significant effect. In multivariable regressions, the factors high age (β = -0.28, 95% CI = -0.48 to -0.08, p = 0.007; see model 5) and high baseline cognitive performance (β = -0.37, 95% CI = -0.58 to -0.17, p = 0.001; see model 5) predicted decrease in cognitive performance, while cardiovascular risk factors (systolic blood pressure, HbA1c, total cholesterol; see model 3), fibrinogen (see model 3), IMT (see model 4) and cardiovascular diseases (CHD, stroke/TIA, PAD, see model 5) were no significant predictors. Since Davey et al. (2013) [23] reported that change of eGFR but not baseline eGFR predicted changes of cognitive performance during the follow-up, we also analyzed how the changes of eGFR, changes of risk factors and changes of atherosclerosis markers, as above, predicted the course of cognitive performance between baseline and follow-up. These analyses did not reveal any links between changes of the above variables and change of cognitive performance, neither in univariate nor multivariable analyses (not shown).

**Table 4:** Baseline Predictors for Change of Global Cognitive Performance from Baseline to Follow-up in the Total Cohort: Unadjusted and Simple Multivariable Models.

	Unadjusted			Model 1			Model 2		
				Corrected R^2^ = 0.005			Corrected R^2^ = 0.087		
	β or B	95% CI	p-value	β or B	95% CI	p-value	β or B	95% CI	p-value
Age	-0.17	-0.35 to 0.01	0.066	-0.16	-0.36 to 0.03	0.104	-0.30	-0.50 to -0.10	**0.004**
Sex (male vs. female)	-0.01	-0.17 to 0.17	0.961	0.02	-0.15 to 0.19	0.848	-0.01	-0.17 to 0.16	0.973
Education	0.02	-0.16 to 0.20	0.823	-0.02	-0.21 to 0.17	0.837	0.06	-0.14 to 0.25	0.572
eGFR	-0.12	-0.30 to 0.06	0.180	-0.10	-0.28 to 0.09	0.300	0.01	-0.19 to 0.19	0.998
Systolic blood pressure	-0.06	-0.24 to 0.13	0.549						
HbA1c	-0.08	-0.26 to 0.11	0.418						
Total cholesterol	0.06	-0.12 to 0.24	0.525						
Fibrinogen	0.10	-0.09 to 0.28	0.295						
IMT	-0.18	-0.36 to -0.01	**0.049**						
CHD, stroke or PAD	-0.15	-0.31 to 0.02	0.079						
Baseline global cognition	-0.21	-0.39 to -0.03	**0.023**				-0.35	-0.56 to -0.14	**0.001**

P-values <0.05 are shown in bold. CHD, coronary heart disease; CI, confidence interval; eGFR, estimated glomerular filtration rate; HbA1c, glycated hemoglobin; IMT, intima-media thickness of common carotid artery; PAD, peripheral artery disease.

**Table 5:** Baseline Predictors for Change of Global Cognitive Performance from Baseline to Follow-up in the Total Cohort: More Complex Multivariable Models.

	Model 3			Model 4			Model 5		
	Corrected R^2^ = 0.041			Corrected R^2^ = 0.083			Corrected R^2^ = 0.109		
	β or B	95% CI	p-value	β or B	95% CI	p-value	β or B	95% CI	p-value
Age	-0.29	-0.52 to -0.07	**0.011**	-0.21	-0.43 to 0.01	0.065	-0.28	-0.48 to -0.08	**0.007**
Sex (male vs. female)	-0.05	-0.22 to 0.13	0.580	0.05	-0.12 to 0.23	0.569	0.03	-0.14 to 0.20	0.708
Education	0.04	-0.17 to 0.24	0.716	0.06	-0.14 to 0.25	0.570	0.02	-0.17 to 0.22	0.803
eGFR	0.08	-0.15 to 0.30	0.498	-0.02	-0.21 to 0.17	0.820	0.01	-0.18 to 0.19	0.944
Systolic blood pressure	0.06	-0.14 to 0.26	0.554						
HbA1c	-0.14	-0.33 to 0.07	0.161						
Total cholesterol	-0.01	-0.21 to 0.19	0.947						
Fibrinogen	0.09	-0.12 to 0.30	0.403						
IMT				-0.18	-0.39 to 0.03	0.093			
CHD, stroke or PAD							-0.17	-0.33 to 0.01	0.053
Baseline global cognition	-0.32	-0.54 to -0.09	**0.006**	-0.32	-0.54 to -0.11	**0.003**	-0.37	-0.58 to -0.17	**0.001**

P-values <0.05 are shown in bold. CHD, coronary heart disease; CI, confidence interval; eGFR, estimated glomerular filtration rate; HbA1c, glycated hemoglobin; IMT, intima-media thickness of common carotid artery; PAD, peripheral artery disease.

## Discussion

In a well-defined cohort of CKD patients that we recruited in a university department of nephrology together with a sample of patients without CKD with very similar vascular risk profile, we herein demonstrate that cognitive performance remains highly stable over a 2-year follow-up. Indeed, cognitive performance did not decrease during the observation period in CKD patients, but rather improved as a consequence of test sophistication and practice [35], which reached significance in the language domain. As a consequence of dedicated patient care, total serum cholesterol and common carotid intima-media thickness significantly decreased during the follow-up, while all other risk factors largely remained unchanged. In multivariable regression studies, high age predicted decrease of cognitive performance during the follow-up period. Until now, the majority of studies examining cognitive performance in elderly subjects with CKD reported deterioration of cognitive performance in longitudinal analyses [36]. In these studies, participants were recruited outside hospital environments and as such many subjects were not clinically ill. Besides, the participants of these studies only partly suffered from CKD. Since cognitive performance related to CKD was not the primary focus of these studies, cognition was assessed by rather simple screening tools, such as the MMSE [11, 12, 18, 19] or 3MS [14] or evaluated only via telephone interviews [13, 15].

The only two studies which used a comprehensive neuropsychological test battery to measure cognitive performance in residential care facility or community-based cohorts reported diverse findings [22, 23]. Notably, only one of these studies reported magnitudes of cognitive changes over time [23]. In 886 dementia-free participants from the Rush Memory and Aging Project cohort, which was recruited in residential care facilities to identify risk factors for Alzheimer's disease, Buchman et al. [22] reported a significant association between low baseline eGFR and decline of cognitive performance. This paper did not provide information about the absolute magnitude of cognitive decline during the follow-up. The follow-up period was slightly longer (3.4±1.4 years) and the cohort was much older (mean age 80.6±7.5 years) than in our study. High age predicted decrease in cognitive performance in multivariable linear regression analyses in our study. The different age of cohorts most likely explains the diverse observations regarding cognitive development in the Rush Memory and Aging Project cohort and the NT^CVD^ cohort.

Similar to our study, Davey et al. [23] could not show an association between baseline eGFR and change in cognitive function in 590 subjects from the community-based Maine-Syracuse longitudinal cohort. In their study, a decline of global cognitive performance of 0.075 z-values was noted over 5 years follow-up, equivalent to a decrease of 0.03 z-values over 2 years. Despite sufficient statistical powering, we did not detect such decline in the present study. The mean age of their cohort was comparable to ours (61.1 years), but the risk factor profile was less severe. The follow-up period of 5 years was considerably longer than in our study. In contrast to our results, the authors observed a significant association between change of log-transformed eGFR and change of cognitive performance when adjusted for baseline cognition, renal function and vascular risk factors. A possible reason for this association, which we could not confirm in our study, is that kidney function was not stable in the Maine-Syracuse cohort, 36.9% of CKD patients experiencing a clinically relevant deterioration of eGFR defined by >3 mL/min/1.73 m²/year [23]. This percentage was significantly lower in our study (16.4% of CKD patients). Differences in the intensity of patient care might explain different observations between the Maine-Syracuse cohort and NT^CVD^ cohort.

The only study that explicitly recruited CKD patients was published by Weng et al. [24]. In a cohort of 125 subjects recruited in a residential care facility, the authors reported no association between kidney function and cognitive decline, which is in accordance with our study. In comparison to our cohort, the participants of this study were much older (78.1±3.6 years). In contrast to our cohort, participants revealed a deterioration of cognitive function independent of eGFR that was stratified at a cutoff of 49 mL/min. The follow-up period in their study (3 years) was similar to our study, but cognitive performance was assessed only using the MMSE and CDR scale, while we used a battery of 10 neuropsychological tests for various cognitive domains that was administered by a clinical neuropsychologist. The different age of participants and the recruitment pathway (residential care facility vs. university hospital) most likely explain differences in the progression of cognitive deficits between this study and the NT^CVD^ cohort.

Based on the combined evidence of the present and the above previous studies, the variables age and disease control may represent the most significant factors modulating cognitive deterioration in patients suffering from CKD. In the NT^CVD^ cohort, multivariable regression analyses also provided evidence that high baseline cognitive performance predicted cognitive decline. Most likely, this effect is attributed to the so-called effect of regression to the mean in subjects with particularly strong or poor baseline test performance [37]. In elderly patients, poor cognitive performance and not good cognitive performance was repeatedly predictive of cognitive decline [24, 38, 39]. Interestingly, in the NT^CVD^ cohort, high IMT, indicative of large-vessel atherosclerosis, and presence of cardiovascular diseases (CHD, stroke/TIA, PAD) exhibited trends for associations with cognitive decline (see Tables 4 and 5). Due to the limited sample size, these associations failed to reach significance in multivariable regression analyses.

The major strengths of this study are the well-defined sample of CKD patients and the detailed neuropsychological examination, which allowed us to detect subtle cognitive changes over time. Due to careful patient investigation, the follow-up rate was very high (83.5%). Despite this fact, biases related to patient dropouts cannot be excluded. The major weakness of this study is its relatively small sample size and the short 2-year follow-up period, which however had an adequate statistical power to detect rates of cognitive decline reported in the past [23] (see paragraph on cognitive performance in the results section). We decided to evaluate this follow-up period since we expected considerably higher dropout rates after longer follow-ups, which may have confounded the conclusions made in this sample of seriously ill patients. In the future, larger studies with longer follow-ups will be required to further dissect the mutual links between CKD and cognitive decline. Considering the impact of clinically overt diseases, there will be a need for clinical multicenter cohorts to evaluate how different treatment strategies may affect the course of cognitive performance.
